# *IN VITRO* INVESTIGATIONS OF *STAPHYLOCOCCUS AUREUS* BIOFILMS IN PHYSIOLOGICAL FLUIDS SUGGEST THAT CURRENT ANTIBIOTIC DELIVERY SYSTEMS MAY BE LIMITED

**DOI:** 10.22203/eCM.v043a03

**Published:** 2022-02-02

**Authors:** S. Isguven, K. Fitzgerald, L.J. Delaney, M. Harwood, T.P. Schaer, N.J. Hickok

**Affiliations:** 1 Department of Orthopaedics, Sidney Kimmel Medical College, Thomas Jefferson University, Philadelphia, PA 19107, USA; 2 Department of Radiology, Sidney Kimmel Medical College, Thomas Jefferson University, Philadelphia, PA 19107, USA; 3 Rothman Orthopaedic Institute, Philadelphia, PA 19107, USA; 4 Comparative Orthopaedic Research Laboratory, New Bolton Center, School of Veterinary Medicine, University of Pennsylvania, Kennett Square, PA 19348, Philadelphia, USA

**Keywords:** *Staphylococcus aureus*, infection, vancomycin, gentamicin, sheep model

## Abstract

Orthopaedic surgical site infections, especially when a hardware is involved, are associated with biofilm formation. Clinical strategies for biofilm eradication still fall short. The present study used a novel animal model of long-bone fixation with vancomycin- or gentamicin-controlled release and measured the levels of antibiotic achieved at the site of release and in the surrounding tissue. Then, using fluids that contain serum proteins (synovial fluid or diluted serum), the levels of vancomycin or gentamicin required to substantially reduce colonising bacteria were measured in a model representative of either prophylaxis or established biofilms. In the *in vivo* model, while the levels immediately adjacent to the antibiotic release system were up to 50× the minimal inhibitory concentration in the first 24 h, they rapidly dropped. At peripheral sites, values never reached these levels. In the *in vitro* experiments, *Staphylococcus aureus* biofilms formed in serum or in synovial fluid showed a 5-10 fold increase in antibiotic tolerance. Importantly, concentrations required were much higher than those achieved in the local delivery systems. Finally, the study determined that the staged addition of vancomycin and gentamicin was not more efficacious than simultaneous vancomycin and gentamicin administration when using planktonic bacteria. On the other hand, for biofilms, the staged addition seemed more efficacious than adding the antibiotics simultaneously. Overall, data showed that the antibiotics’ concentrations near the implant in the animal model fall short of the concentrations required to eradicate biofilms formed in either synovial fluid or serum.

## Introduction

To minimise SSIs, orthopaedic surgeons routinely administer antibiotic prophylaxis (Parvizi et al., 2017; Sweet et al., 2011), especially in revision surgeries (Jiranek et al., 2006). However, the challenge of local, perioperative, prophylactic antibiotics is the lack of consensus on the amount, timing, release profile and type of antibiotic that would be most beneficial, resulting in a high variability in antibiotic prophylaxis (Chen et al., 2018; Kamath et al., 2016). Utility of perioperative prophylaxis, such as systemic administration of cefazolin or VAN is agreed upon (Parvizi et al., 2017). While the increased benefit of local antibiotics in the absence of a clinical infection remains controversial (Jiranek et al., 2006; Parvizi et al., 2017), local antibiotic prophylaxis continues to be performed, *e.g.* the placement of a VAN powder during wound closure when performing spinal implant surgery (Khan et al., 2014; Lin et al., 2021) or the use of drug-loaded local delivery systems such as GEN sponges (Han et al., 2016). In all cases, antibiotic prophylaxis is designed to eradicate the perioperative contaminants that have the potential to progress to SSIs.

The choice of antibiotics, especially for prophylaxis, is guided by the principles set forth by the American Academy of Orthopaedic Surgeons, enhancing the surveillance of three quality measures related to infection prevention, namely that patients: 1) receive prophylactic antibiotics consistent with current recommendations; 2) receive prophylactic antibiotics within 1 to 2 h prior to surgical incision (Bratzler et al., 2013); 3) have prophylactic antibiotics discontinued within 24 h following the end of surgery (Berbari and Baddour, 2020; Hansen et al., 2013). These guidelines largely consider systemic and intravenous administration of antibiotics rather than locally administered and contained drugs. The selected antibiotics are dictated by the fact that *S. aureus* and the coagulase-negative Staphylococci are the most common causes of orthopaedic infections (Chirca and Marculescu, 2017; Rao et al., 2008). *In vitro* and *in vivo*, *S. aureus* rapidly forms biofilms and bacterial aggregates/floating biofilms in wound fluid and SynF. These biofilms show increased antibiotic tolerance (Costerton et al., 1999) and, in physiological fluid, tolerance may increase (Dastgheyb et al., 2015b; Gilbertie et al., 2019). Thus, increased local delivery/presence of antibiotics remains a possible solution (Adams et al., 2009; Jiranek et al., 2006; Parvizi et al., 2017; von Plocki et al., 2012; Schwarz et al., 2021) for both prophylaxis and established infections.

In patients presenting clinical signs of a PJI, DAIR is often attempted to avoid more invasive interventions required with a one- or two-stage implant exchange (Cobo et al., 2011; Masters et al., 2019). Local antibiotic therapy is provided by antibiotic-impregnated PMMA cement spacers or beads, biodegradable polymers or regional limb perfusions (Kanellakopoulou and Giamarellos-Bourboulis, 2000; Zalavras et al., 2004). However, the rapid elution kinetics of carrier systems result in antibiotic levels that often drop below the MIC and allow the porous, non-degradable carrier matrices to become a substrate for bacterial adherence and biofilm formation (Neut et al., 2001). Antimicrobial tolerance and even fostering of resistance in the presence of sub-MIC antimicrobial concentrations may further complicate successful resolution of the infection (Smith, 2005). Importantly, even when *S. aureus* is added to synovial fluid containing many times the MIC of prophylactic antibiotics, bacterial eradication is attenuated (Dastgheyb et al., 2015b), suggesting that MIC is inadequate for predicting eradication. The situation is only worsened in established infections where a biofilm is present. Some researchers have attempted to address this issue by investigating values such as MBEC (Sepandj et al., 2004). Importantly, a higher concentration of antibiotics may be required not only to treat existing biofilms but to prevent bacterial adhesion in the first place.

The present study sought whether the concentrations of antibiotics that were eluted from local elution systems *in vivo* would be sufficient to markedly decrease, if not eradicate, *S. aureus* biofilms, *in vitro*. The study was performed using either a system that mimics another elution system [such as the TRYX Absorbable Antibacterial Envelope (Medtronic, Minneapolis, MN, USA) (Tarakji et al., 2019)] or that contain a bioglass, as used for drug delivery in bone (Baino et al., 2016; Soundrapandian et al., 2010). Specifically, the study analysed local ECF data generated from two different antibiotic elution systems, one in an IM site and the other adjacent to the bone, such as would be used in a fracture plate. From these ECF samples, the concentrations and duration of antibiotic elution at the site of implantation, as well as in surrounding tissues, were determined. Using these antibiotic ranges as a guideline, the study explored concentrations of VAN and GEN, separately and together, that would eradicate 24 h biofilms or prevent bacterial colonisation on the Ti6Al4V alloy and PLA. Finally, the study assessed the utility of a staged combination delivery of VAN and GEN, as antibiotic combinations have been recommended to reduce the risk of resistance (Brooks and Brooks, 2014). The study findings raised questions about the requirements for effective local concentrations of antibiotics against biofilms.

## Material and Methods

### Ethics statement

Anonymised human synovial fluid samples from therapeutic joint aspirations were retrieved and designated as “waste” and “not human research” by the Thomas Jefferson University Institutional Review Board, as per the revised Common Rule (2018). The IACUC of the University of Pennsylvania approved the ovine study following ARRIVE guidelines (du Sert et al., 2018).

### *In vivo* pilot studies

Skeletally mature sheep were enrolled in a pilot study of a non-infected long-bone (tibia) model with an implanted hardware to determine actual antibiotic concentrations at both the elution system site (GEN = fracture fixation plate, VAN = IM Ti alloy rod) and at sites distant from the eluting antibiotics.

Skeletally mature, healthy, female Dorset crossbred sheep, with unlimited access to exercise, were acclimatised for 14 d prior to study enrolment. The day before surgery, based on a detailed physical examination, sheep free of signs of clinical disease were fasted (24 h) and allocated to VAN_IM_ (*n* = 4), GEN_Plasma_ (*n* = 8) or GEN_ECF_ (*n* = 3) study cohorts. On the day of surgery, a left jugular catheter was inserted under aseptic conditions. Following sedation using diazepam (0.5-1.5 mg/kg, IV), anaesthesia was induced using ketamine (2.2-4.0 mg/kg) and animals were endotracheally intubated and placed in dorsal recumbency. Anaesthesia was maintained using isoflurane (1.25-5 %) in oxygen and animals were monitored using standard equipment supported by a jugular venous catheter, arterial line (blood pressure, arterial blood gas), pulse-oximetry, ECG, FIO_2_, CO_2_. If necessary, animals were mechanically ventilated. No perioperative antimicrobial prophylaxis was provided. Analgesia was provided for 3 d perioperatively and consisted of transdermal fentanyl patches (2.5 μg/kg/h) placed 12 h prior to surgery and left in place for 72 h and intravenous flunixin meglumine (1.1 mg/kg, eveiy 12-24 h). Analgesia was continued based on daily pain assessment by a veterinarian. Using aseptic technique, 11 animals underwent a transverse mid-diaphyseal tibial osteotomy and unilateral plating (9-hole commercially pure Ti LCP, DePuy Synthes) and 3 animals received a non-structural intramedullary Ti alloy rod (Ti6Al4V) coated with VAN and placed in anterograde fashion *via* the tibial plateau. The IM rod was interlocked proximally using one 3.5 mm cortex screw. Animals in the VAN cohort did not receive a tibial osteotomy. During recovery, the operated limb was splinted with a bi-valve fibreglass splint to provide protection during recovery, during transport and until the animal settled in postoperatively (up to 48 h). After completion of surgery, each animal was observed until it was able to stand and walk to a stall. After complete recovery, it was returned to its housing. Perioperative analgesia (2.5 μg/kg/h fentanyl patches) was administered for a period of 72 h (longer if signs of pain were apparent). Clinical scores reflecting pain and animal welfare were recorded daily for 2 weeks following each surgery, then weekly until sacrifice.

#### Drug carrier matrices: GEN antimicrobial sleeve

The tightly adhering, perforated envelope covering the LCP was made of polyglytone 6211^™^, a bioabsorbable 87 lactide-glycolide-trimethylene carbonate-caprolactone polymer. This thin envelope was cast using approximately 43 mg of anhydrous GEN sulphate, which remained in a slurry (because of limited solubility) in the polymer solution and was fabricated so that it slipped over the LCP (Synthes), as previously described by von Plocki et al. (2012).

#### Drug carrier matrices: VAN

The VAN coating of the IM nail consisted of multiple coatings of bioactive glass with a nominal VAN concentration of 20 wt % (percent of drug weight to SiO_2_ weight) (Adams et al., 2009). Briefly, IM nails comprised of the Ti6Al4V alloy (length: 140 mm; diameter: 6 mm) were sandblasted, cleaned and dried. The sandblasted and cleaned nail (substrate) was further sonicated in acetone for 30 min and 2 % detergent for 1 h and finally rinsed with DI water. Then, a fresh oxide coating was created by passivation in 35 % nitric acid for 1 h, followed by rinsing with DI water and drying in a laminar flow hood. For the coating procedure, a dipping device with controlled speed and mechanics was used, to ensure even deposition of layers. Each layer was comprised of the sol-gel film containing 20 wt % VAN and was dried in a laminar flow hood for 2 h before application of the next layer for a total of 10 layers. After application of the last layer, the films were dried overnight (Adams et al., 2009).

#### Ultrafiltration probes

**Fig. 1.**
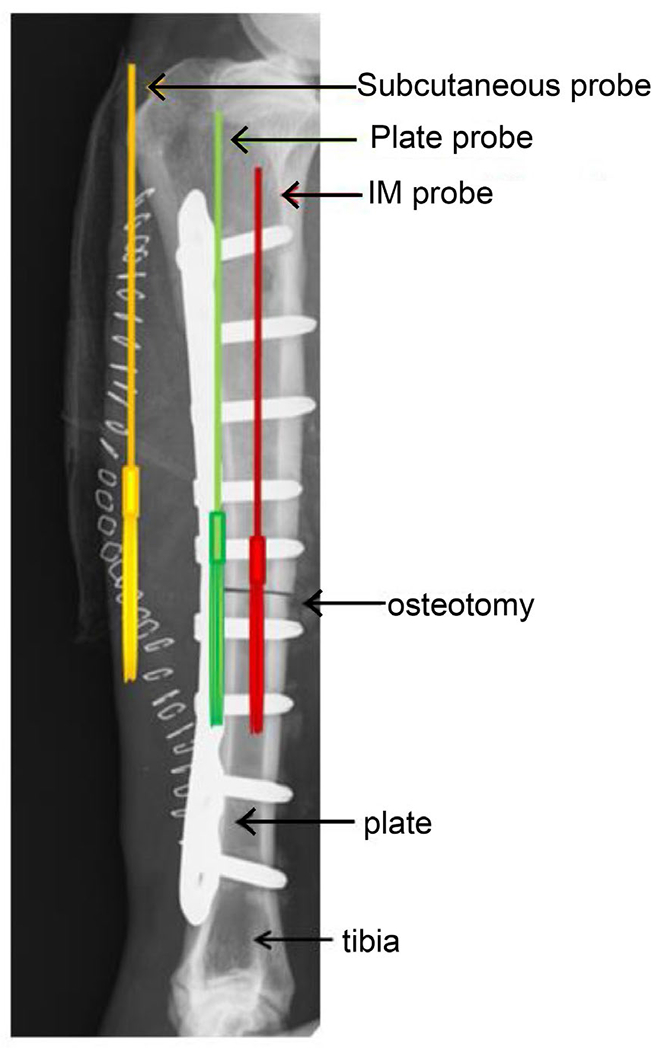
Probe placement in sheep long bone. The radiograph shows the LCP with GEN delivery system (not visible in radiograph), osteotomy and the three ultrafiltration probes in the three distinct tissue compartments: IM canal (red), periosteal surface (green) and soft tissue envelope (yellow). The ultrafiltration probes for the VAN-coated Ti alloy rod were placed in the same fashion.

All animals were fitted with customised ultrafiltration probes (30 kDa), supplied by BASInc (West Lafayette, IN, USA) and fitted with a protective outer polyurethane sleeve to improve durability. Probe distribution was IM (mid-diaphysis), PO (mid-diaphysis) and SQ (mid-diaphysis) (Fig. 1). Probe tubing was tunnelled subcutaneously towards the lateral aspect of the femur and externalised collection vials were supported by a tight fitting blanket. Collection vials were checked at 1, 2, 4, 6, 8, 10, 12 and 24 h post-operative and then daily for the duration of the 30 d study. All tubes were replaced at each time point. Fluid was collected from tubes that contained the minimum volume (> 0.1 mL) for analysis of local tissue concentration of GEN or VAN.

#### Drug analysis

Venous blood samples (K_2_EDTA sheep plasma) were taken at time – 0 (immediately prior to antibiotic-impregnated carrier matrix placement), + 0 (immediately following carrier matrix placement) and at probe-sampling times for the first 10 d postoperative. After this time, venous blood samples were obtained every 7 d until sacrifice, and at end term prior to euthanasia. For ECF collection, GEN or VAN samples were obtained following the same sampling schedule as described above.

#### LC/MS/MS analysis of GEN and VAN

100 μL of a GEN or VAN sample was mixed by vortexing with 200 μL of an internal standard solution (tobramycin at 250 ng/mL in methanol containing 0.1 % formic acid) in a capped 96-well Nunc polypropylene plate, followed by centrifugation (1,500 ×*g* for 10 min). Then, a 150 μL aliquot of the supernatant was mixed by vortexing with 150 μL of water/0.1 % formic acid in a sterile, capped 96-well Nunc polypropylene plate, followed by centrifugation (3,000 ×*g* for 5 min). This extract was injected onto a HPLC system equipped with a triple quadrupole tandem mass spectrometer (AB/MDS Sciex API-5000) detector operated in positive TurboIonSpray^®^ mode. GEN or VAN were separated from extracted matrix materials using a Varian Pursuit C18 XRs column (50 × 2.0 mm, 3 μm particle size) at room temperature using a gradient mobile phase system of 0.2 % heptafluorobutyric acid in water (mobile phase A) and 0.1 % heptafluorobutyric acid in acetonitrile (mobile phase B) at a total flow rate of 300 μL/rnin. Calibration standards, prepared fresh daily at 10.0 to 2,000 ng/mL, were used to construct standard curves for GEN or VAN.

### *In vitro* studies

#### Materials

Machined Ti6Al4V (10 × 2 mm, kind gift of Zimmer Biomet) and 3D-printed PLA (Ultimaker, 1.24 specific gravity, 10 × 2 mm) were used. Ti6Al4V was cleaned using 4 mol/L HNO_3_. Then, Ti6Al4V and PLA were rinsed with distilled (DI) water, sonicated in 70 % ethanol for 15 min and sterilised under UV light for 20 min. Samples were stored sterile and dry until inoculation.

#### Bacterial strains and growth

A single colony of MSSA ATCC^®^ 25923^™^ was grown in TSB (Becton-Dickinson) ON at 37 °C and 180 rpm, subcultured for 2-3 h and diluted by comparison to a 0.5 McFarland standard (~ 10^8^ CFU/mL for MSSA). MSSA ATCC^®^ 25923^™^, known for its biofilm-forming capacity (Goggin et al., 2014), was used as a reference strain (Treangen et al., 2014). Strain integrity was achieved by using frozen subcultures from the commercially available ATCC^®^ strain, periodic culturing on blood agar plates to test for haemolysis (Wiseman, 1975) and on selective maltose salt agar. Strain maintenance is ensured by periodically measuring the antibiotic sensitivity using Etest strip (Biomerieux, Marcy-l’Étoile, France) and MIC experiments (ATCC25923: VAN = 2μg/mL; GEN = 0.25 μg/mL by Etest).

#### Bacterial adhesion and biofilm antibiotic treatments

Biofilms were pre-formed in a 24 h static culture before antibiotic was added or bacteria and antibiotics were added in a simultaneous fashion. For preformed biofilm experiments, Ti6Al4V and PLA discs were submerged in 1.0 mL TSB or SynF [using 24 well tissue culture plates (Med Supply Partners, Atlanta, GA, USA)], inoculated with 10^5^ CFU/mL MSSA and incubated for 24 h at 37 °C. Then, the resulting surfaces were incubated with 0-500 μg/mL VAN (Athenex, Buffalo, NY, USA) for 24 h at 37 °C in TSB or human synovial fluid. For simultaneous addition experiments, the Ti6Al4V and PLA discs were submerged and bacteria added using TSB, SynF, 50 % serum (both human and foetal bovine, Sigma-Aldrich)/TSB or 50 % serum/PBS (MP Biomedicals, Santa Ana, CA, USA). 0-100 μg/mL GEN (Alfa Aesar, Haverhill, MA, USA) or 0-100 μg/mL VAN were added at indicated concentrations and times (immediately after inoculation to up to 30 min after inoculation) and incubated for 24 h at 37 °C. Surfaces were gently washed with PBS to remove planktonic bacteria and adherent bacteria were resuspended by bath sonication for 15 min at 40 kHz in 0.3 % Tween 20/PBS. Suspended bacteria were serially diluted, plated on 3M^™^ Petrifilm^™^ (aerobic count, 3M Corporation), incubated at 37 °C for 24 h and counted (countable range, 30-300 CFU/spot).

#### Checkerboard assay for antibiotic synergy

96-well tissue culture plates (Med Supply Partners), containing 10^5^ CFU/mL MSSA in MHB were tested using a matrix of VAN (0-8 μg/mL) and GEN (0-4 μg/mL), with antibiotics added together or 20 min apart. FIC index was calculated using MIC, following the equation

$$\text{A}/{\mathrm{MIC}}_{\text{A}}+\text{B}/{\mathrm{MIC}}_{\text{B}}=\text{FIC index}$$

where A and B are VAN and GEN concentrations in a single well, respectively. FIC index < 0.5 denotes synergy, > 4 antagonism, 0.5-4 additivity (Meletiadis et al., 2010).

#### SEM

Samples were fixed using 4 % paraformaldehyde (buffered in PBS) at RT for 15 min and dehydrated by sequential incubation (RT, 10 min) with 10 %, 30 %, 50 %, 70 %, 90 % and 100 % ethanol in DI water. Samples were air-dried in the fume hood ON, sputter-coated (Cressington 108 Auto Sputter Coater, Pella, Inc., Reading, CA, USA) with gold or platinum/palladium for approximately 20 s and imaged using a Hitachi TM-1000 SEM with an accelerating voltage of 15 kV.

### Statistics

#### In vitro

3 separate experiments, each containing 6 independent determinations were performed. For simple comparisons between two populations, statistical significance was determined using the Student’s *t*-test or Mann-Whitney U test, based on the normality of data. For multiple comparisons, for normally distributed data, a one-way ANOVA with Bonferroni correction and Tukey’s multiple comparison *post-hoc* test was used, with an Alpha value of 0.05; for nonparametric comparisons, a Kruskal-Wallis test with Dunn’s multiple comparison correction was used (GraphPad Prism ver 8.4.0).

#### In vivo

For the plasma, GEN or VAN data (C_max_, T_max_, AUC and max concentration) were calculated. Linear weighted (1/×2) regression analysis of peak area ratio *versus* theoretical concentration was used to produce the calibration curves.

## Results

### *In vivo* sampling of ECF using ultrafiltration probes

The *in vivo* experiments measured the local tissue distribution of antibiotics over time when placed as a delivery system in a clinically relevant location. All sheep in the pilot study had uneventful recoveries from surgery and general anaesthesia and completed the study. Vacutainers (Fig. 1) and probe tubing remained in place throughout the study and were well tolerated by all animals.

Local ECFs to determine GEN and VAN concentration were collected at 1, 2, 4, 6, 12 and 24 h after probe placement, then daily thereafter for up to 30 d. During the first 24 h, sample volumes were variable, ranging from 0.1 to 1.0 mL for the IM probes, from 0.2 to 1.7 mL for the plate probes and from 0.1 to 1.3 mL for the SQ probes. After the 24 h timepoint, the mean volume per collection time point was 1.4 mL for SQ probes, 1.0 mL for plate probes and 0.2 mL for IM probes. In the VAN sheep cohort, ECF volumes sufficient for analysis were unable to be reliably collected after day 14 due to tubing tortuosity. This was corrected for the GEN sheep cohort so that sufficient volumes were collected up to 28 d postoperative.

### Local VAN ECF and plasma concentration

**Fig. 2.**
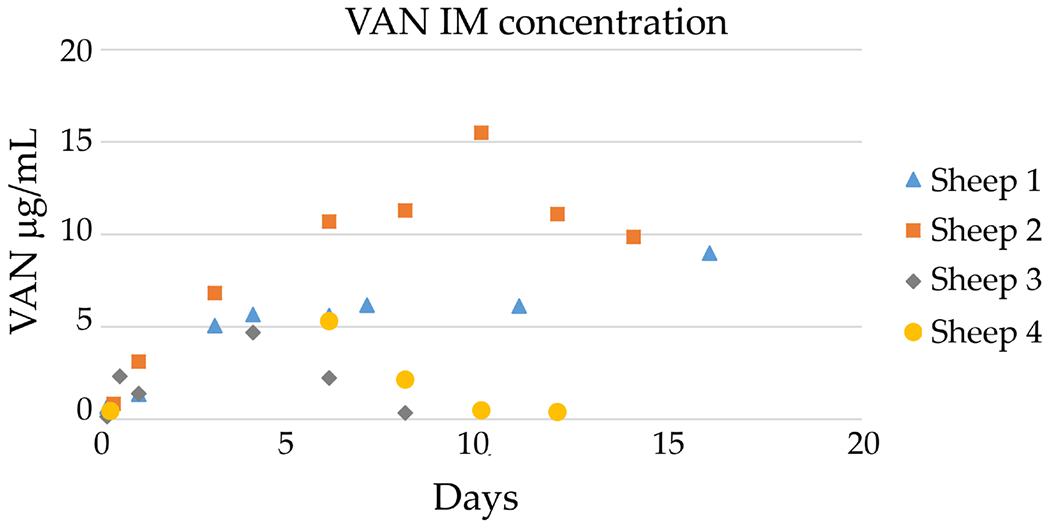
VAN concentrations over time at the site of antibiotic elution. ECF VAN concentration at each sampling interval from the IM ultrafiltration probe location: sheep 1: C_max_ 8.98 μg/mL, T_max_ 16 d; sheep 2: C_max_ 15.50 μg/mL, T_max_ 10 d; sheep 3: C_max_ 4.69 μg/mL, T_max_ 4 d; sheep 4: C_max_ 5.30 μg/max, T_max_ 6 d.

When VAN elution from the coated Ti alloy rod was measured, the local VAN concentration rapidly increased in all 4 animals over the first 4 d. Then, 2 animals exhibited a drop-off in VAN concentration, whereas the remaining 2 showed peak VAN elution at 10 d. The maximum VAN concentration achieved in the IM canal was C_max_ 15.50 μg/mL in sheep 2 at T_max_ 10 d postoperatively (Fig. 2). In sheep 1, VAN IM concentration first plateaued at around 6 μg/mL followed by a second release of C_max_ 8.98 μg/mL, T_max_ 16 d. Both sheep 3 and 4 showed a C_max_ around 5 mg/mL at 4 and 6 d, respectively. On the other hand, the plasma, SQ and plate VAN concentrations remained below the LC/MS/MS detection limit (0.050 μg/mL) in all sheep. Notably, even at peak concentrations, local VAN did not exceed 5-15× MIC for MSSA.

### Local GEN ECF and plasma concentration

**Fig. 3.**
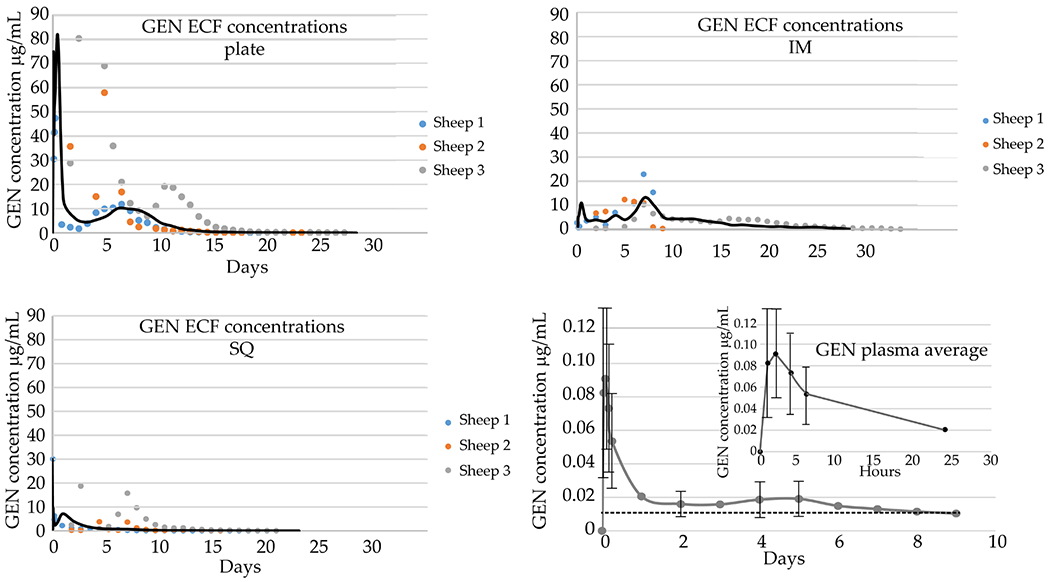
GEN ECF and plasma concentrations over time. GEN ECF concentrations at each sampling interval from 3 ultrafiltration probe locations: IM cavity, SQ, periosteal/adjacent to plate (plate). Each ECF graph shows data from individual sheep as well as the average plotted as a continuous black line. IM probe: maximum mean concentration (C_max_) 13.1 μg/mL, time of C_max_ (T_max_) 7 d. Plate probe: C_max_ 80.5 μg/mL, T_max_ 12 h. SQ probe: C_max_ 30.0 μg/mL, T_max_ 0 h. Plasma GEN concentration at each sampling interval with a C_max_ 90.5 ng/mL, T_max_ 2 h. Values from 8 animals were used to determine the shape of the elution curves. The dotted line represents the limit of detection, 0.010 μg/mL, and only data points that were above this detection limit were used in the analysis. The error bar is missing for 24 h time point as *n* < 3 samples due to the detection limit.

Using the GEN polymer system (Fig. 3; total GEN = 43 mg), the maximum mean GEN concentration at the plate was 80.50 μg/mL [0.5 d (12 h)]; 13.1 μg/mL peak mean concentration was measured in the IM cavity at 7 d (IM). The drug concentration of 9.49 μg/mL in the soft tissue envelope was the peak mean concentration measured at 2 h (SQ). In these graphs both the lines resulting from plotting i) the mean of the concentrations and ii) the individual values from the different animals are represented. Notably, IM and SQ trends reflected a rapid elution in most animals during the first several days, whereas the plate concentrations showed variability.

The plasma GEN concentration-time curve followed the trend of the GEN levels for the plate probe but the C_max_ of 0.09 μg/mL at T_max_ 2 h was markedly lower (Fig. 3). Specifically, C_max(plasma)_ Was decreased by approximately 100× when compared to the drug concentration obtained from the SQ and IM probe samples and by 1,000× when compared to the plate probe samples. The plasma GEN concentration was below reported safe blood levels of less than 2 μg/mL (Dahlgren et al., 1975).

Based on the elution data, at its maximum, the local concentration of GEN reached ~ 80 μg/mL within the first 24 h. The maximum local concentration of VAN was ~ 15 μg/mL over the course of 10 d.

### Ti6Al4V and PLA surface and biofilm observations in SEM

**Fig. 4.**
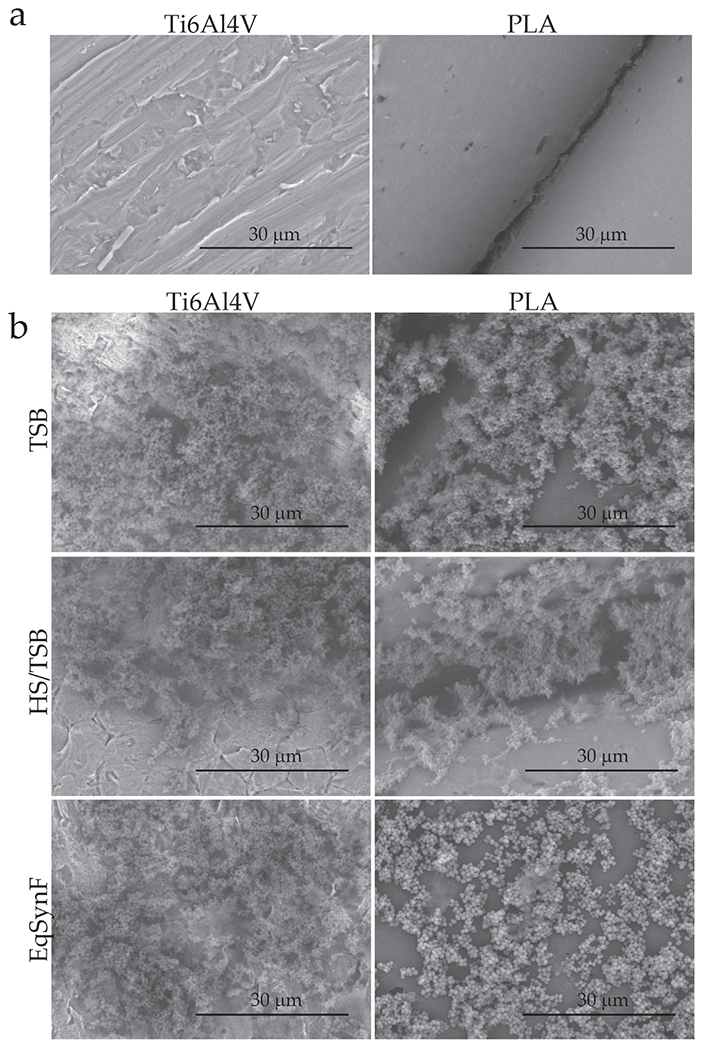
Dry surface and biofilm morphology in different media. (**a**) Dry, sterile Ti6Al4V and PLA surface structures as visualised by SEM. (**b**) Biofilms (24 h) formed in TSB, HS/TSB or EqSynF on Ti6Al4V or PLA surfaces, as visualised by SEM.

To determine the effects of these ranges of concentrations on adherent bacteria *in vitro*, the effects of different media and surfaces on biofilm formation were determined using SEM. The bare Ti6Al4V discs showed machining lines, as well as surface features consistent with minor abrasions during nitric acid cleaning(Fig. 4a), whereas the PLA was 3D-printed so that the filament surface and melted interface were visible. When *S. aureus* was grown on either surface in TSB, HS/TSB or eqSynF, abundant bacterial colonisation was apparent, with abundant 3D structures independent of medium or surface (Fig. 4b). In TSB, colonies were visible in their entire spherical shape, with only a small percentage of the colonies embedded in mucinous extracellular matrix. In HS/TSB and eqSynF images, the mucinous nature of the biofilm was more visible. Specifically, in the PLA, HS/TSB image, small fibres that organise the structure were apparent, with areas covered by a matrix so that individual colonies were obscured. Bacteria grown in eqSynF [SynF is a filtrate of blood (Felgenhauer and Hagedorn, 1980)] on both surfaces were similar to those on HS/TSB in terms of fibrous connectivity and mucinous matrix.

### VAN tolerance of preformed MSSA biofilms as a function of surface and media

**Fig. 5.**
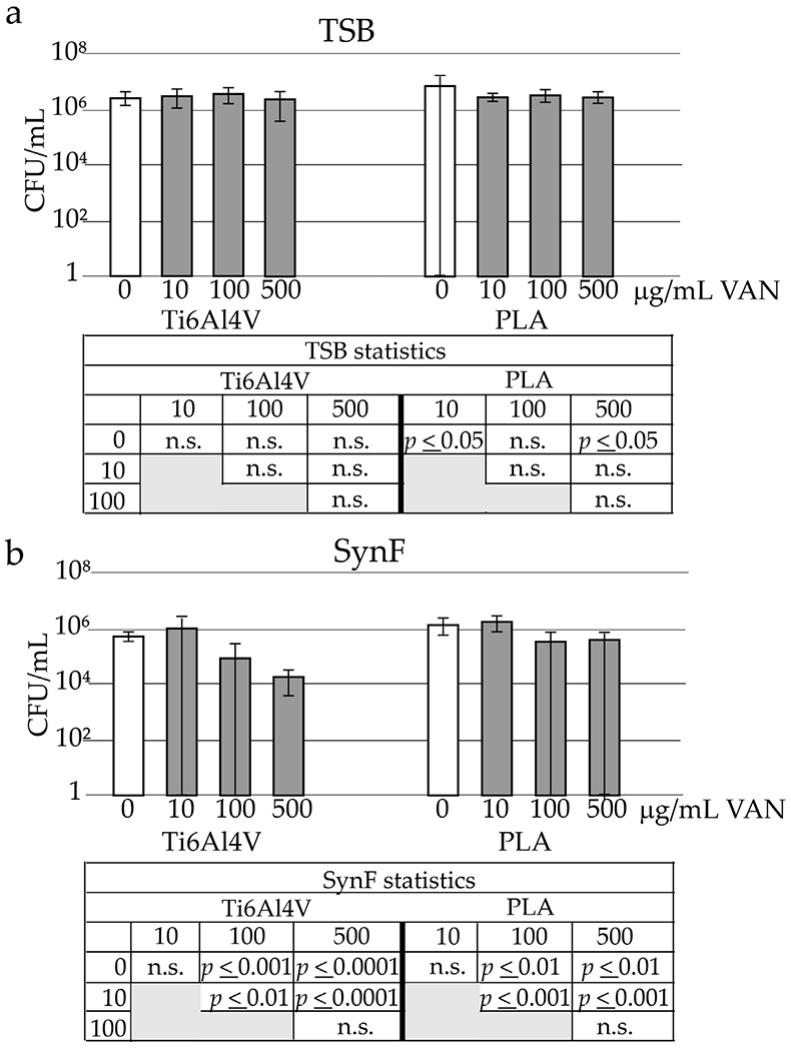
VAN tolerance of biofilms in TSB and SynF. Effects of increasing doses of VAN on bacterial number in 24 h biofilms as a function of medium (**a** TSB, **b** SynF) and surface. *n* = 18 independent observations per data point. Statistical determinations are tabulated below the figures. n.s. = not significant.

Active infection, characterised by surfaces covered with biofilm, was induced. VAN tolerance of preformed biofilms depended on the medium (Fig. 5). In TSB, up to 500 μg/mL VAN showed no significant effect on MSSA in a 24 h biofilm formed on a Ti6Al4V surface (Fig. 5a). For biofilms formed in TSB and on PLA surfaces, 10 μg/mL and 500 μg/mL VAN were statistically different from the control, while 100 μg/mL VAN was not. However, the values were not biologically significant despite their statistical significance, even at 500 μg/mL VAN. Bacterial killing in SynF biofilms showed a small (< 2 log) decreases at the 100 and 500 μL concentrations (Fig. 5b) on both Ti6Al4V and PLA. These values were statistically different from control and from each other.

### Concomitant addition of VAN with MSSA

**Fig. 6.**
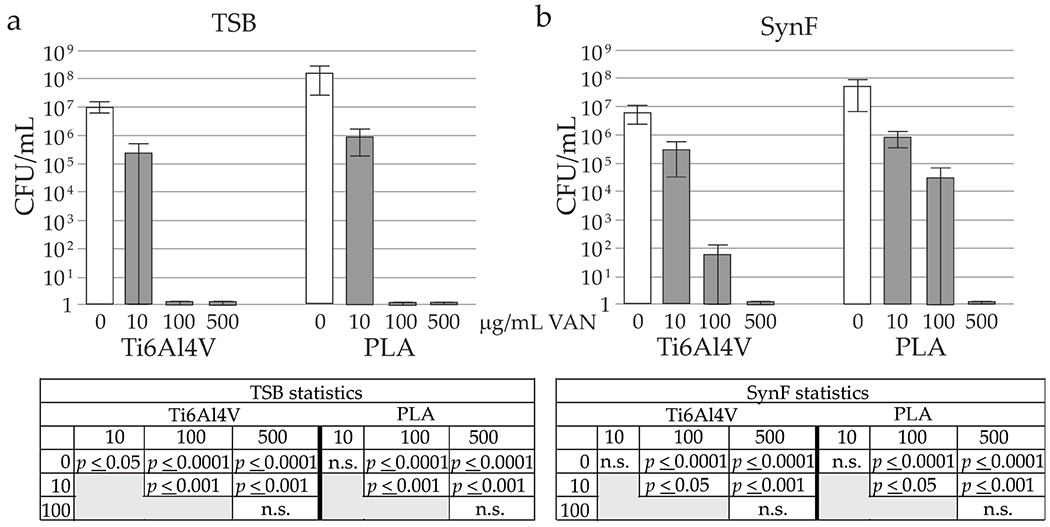
VAN dose response on surfaces. Number of adherent bacteria 24 h after concomitant addition of VAN and 10^5^ CFU/mL in (**a**) TSB (*n* = 18) or (**b**) SynF (*n* = 12). Statistical determinations are shown for the different media and surfaces below the histograms, with n.s. = not significant.

Bacterial contamination was performed in the presence of antibiotics. In TSB (Fig. 6a), 10 μg/mL VAN significantly decreased MSSA numbers, with complete eradication by 100 μg/mL, independent of surface. In SynF (Fig. 6b), 10 μg/mL VAN significantly decreased bacterial colonisation. At 100 μg/mL, this decrease was more marked on the Ti6Al4V surface compared to the PLA (both surfaces, *p* < 0.0001 compared to control) (Fig. 6b). Complete eradication only occurred at 500 μg/mL (*p* < 0.0001 compared to control for both surfaces).

### SynF and serum effects on antibiotic tolerance

**Fig. 7.**
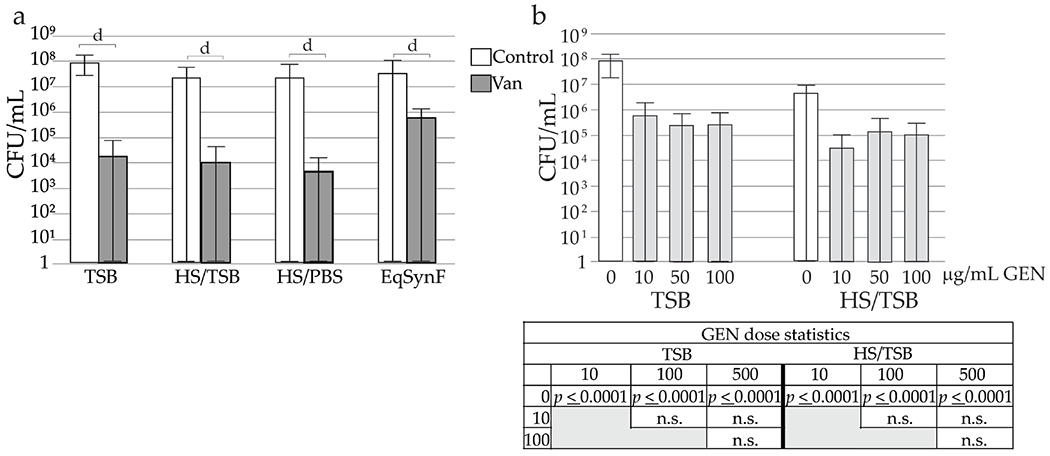
Effects of media and concentration on VAN and GEN tolerance. (**a**) Comparison of different medium on the number of adherent bacteria retrieved (24 h) after concomitant addition of 10 μg/mL VAN and MSSA; *n* = 27 for each condition. Control and VAN comparison conducted only within each type of media. ^*d*^ *p* < 0.0001. (**b**) Antibiotic efficacy of GEN when added simultaneously. Numbers of adherent bacteria are shown (*n* = 24 for TSB, 18 for HS/TSB). Statistical determinations are shown for the different media and surfaces below the histogram, with n.s. = not significant.

Because both serum and SynF are rich in serum proteins, MSSA adhesion was compared on PLA in TSB, 50 % HS with TSB (HS/TSB), 50 % HS with PBS (HS/PBS) and eqSynF, when the bacteria and 10 μg/mL VAN were added simultaneously (Fig. 7a). In the absence of antibiotics, similar average numbers of MSSA were adherent for all four media. When 10 μg/mL VAN was added, average numbers of adherent bacteria decreased by ~ 4 logs in TSB, HS/TSB and HS/PBS. However, VAN in eqSynF showed attenuated killing (1.5-2 logs). In the presence of antibiotics, numbers of adherent bacteria were highly variable in all media, partially due to the averaging of 5 experiments to give 27 values/determination. Overall, the trends showed that VAN was less effective on average in eqSynF than in other media.

For measuring the dose dependence of GEN, TSB and HS/TSB were used. HS/TSB was chosen to supply proteins present in wound fluid. Simultaneous addition of GEN with MSSA resulted in significant decreases in adherent MSSA at all doses compared to control, both in TSB and HS/TSB (*p* < 0.0001) (Fig. 7b). However, increasing GEN doses did not cause larger decreases in bacterial numbers on average.

### Combined VAN and GEN against adherent and planktonic MSSA

**Fig. 8.**
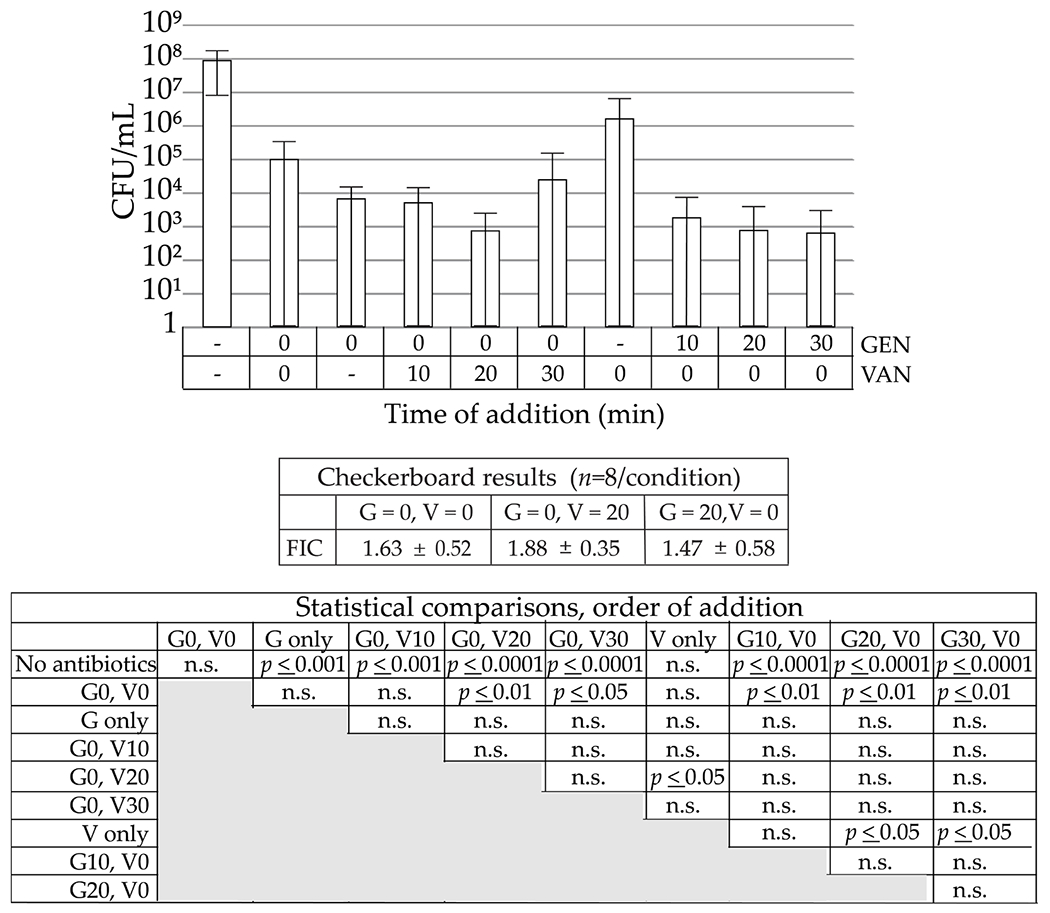
Staged addition and effects on biofilm. The X-axis labels denote the time of addition of each antibiotic, GEN = 10 μg/mL, VAN = 10 μg/mL; *n* = 30. The FIC scores are presented for the same combinations on planktonic MSSA (*n* = 8). Statistical determinations are shown below the FIC scores, with n.s. = not significant.

Addition of GEN or VAN alone (10 μg/mL each) or any combination of GEN + VAN caused a decrease in adherent bacteria compared to no antibiotics and these comparisons reached statistical significance except for control *vs*. VAN only or control *vs*. simultaneous addition of GEN and VAN (G0, V0) (Fig. 8). Simultaneous addition of GEN + VAN did not cause a significant increase in the antibacterial activity over that of GEN or VAN alone. Trends for the staged additions suggested an increased activity and staged additions were statistically significant compared to no antibiotic control as well as compared to G0, V0, except for G0, V0 *vs*. VAN added 10 min after GEN (G0, V10). To further investigate possible additivity of GEN + VAN, antibiotic synergy was determined performing the checkerboard assay using planktonic bacteria. GEN + VAN, independent of time of addition, showed some additivity, but not synergy.

## Discussion

Prevention of bacterial adhesion to implanted materials and subsequent biofilm formation are major concerns, especially when materials are implanted in a site that has had an SSI (Ricciardi et al., 2018). Development strategies for eradication rely on local drug delivery systems driven by strain MIC guidelines (Stebbins et al., 2014; Syal et al., 2017). The present study described a new probe system that allowed measurements of eluted drugs at the implant site and within adjacent tissues, in a sterile, long-bone sheep model. Based on the measured elution rates of 5-50× MIC for VAN and 10-50× MIC for GEN (both determined for MSSA) (Leonard et al., 2013; Wang et al., 2006), the effects of these ranges of VAN and GEN on reduction of bacterial adhesion were determined in an *in vitro* model. Based on the *in vitro* analyses, it was concluded that eluted antibiotics in this range were only moderately effective at preventing bacterial colonisation, especially when cultured in the presence of SynF or 50 % HS.

While VAN- or GEN-loaded carrier systems are widely used to treat osteomyelitis associated with infected fracture sites and PJIs (Schwarz et al., 2021), there are limited *in vivo* studies *vs*. the many *in vitro* elution determinations (Henry and Galloway, 1995). These studies are further limited by questions about superior efficacy over parenteral antibiotic therapy (Diaz-Ledezma et al., 2014) and optimal dosing regime and duration of therapy for orthopaedic infections (Schwarz et al., 2021). Antibiotic-impregnated PMMA cements or biodegradable polymers are used for local antibiotic delivery (Garvin and Feschuk, 2005; Kanellakopoulou and Giamarellos-Bourboulis, 2000; Rutledge et al., 2003; Stewart, 2002; Wininger and Fass, 1996; Zalavras et al., 2004) and the Musculoskeletal Infection Society consensus states that (1) an antibiotic-impregnated cement reduces incidence of PJIs following elective revision joint arthroplasty and that (2) antibiotics should be added to a cement in all patients undergoing cemented or hybrid fixation as part of a revision arthroplasty (Diaz-Ledezma et al., 2014). Although use of antimicrobial-impregnated PMMA is recommended by the East Practice Management Guidelines Workgroups, adequate tissue levels of antimicrobials may not be achieved without additional systemic antimicrobials (Luchette et al., 2000). An abiding concern in all elution systems is the elution kinetics, where the rapid antibiotic release allows antibiotic levels to drop below MIC levels, raising the spectre of the antimicrobial resistance (Gullberg et al., 2011; Smith, 2005).

Animal models provide an elegant means to analyse local tissue concentrations of antibiotics over time (Orsini et al., 1992; Stolle et al., 2008; Thomassen et al., 2020). Previous studies have included a porcine model of local elution, which used IM microdialysis accompanied by serial bone samples (Stolle et al., 2008; Thomassen et al., 2020), and a canine model with serial aspiration of seroma from surgical sites for analysis of local tissue concentrations of antibiotic over time (Adams et al., 1992). As both studies required anaesthesia for continued collection, neither allowed ambulation and the influence of biomechanical load, including gravity on fluid dynamics. The present pilot study, placing collection probes at key tissue sites, allowed the measurements without anaesthesia and for long times. However, there was variability in the antibiotic measurements, as not all samples obtained were of sufficient volume.

As a priority, the present study established a robust methodology to sample ECF from a region of interest (*i.e.* tibia and soft tissue envelope) in an ambulating model over a clinically relevant time. In the pilot study, plasma concentrations of VAN and GEN demonstrated levels consistent with values indicated in the literature for VAN prophylaxis (Yusuf and Croughs, 2020). C_max_ values measured for the IM canal and fracture plate site were in the 10-100× MIC range for staphylococcal species, consistent with other reports (Gustafson et al., 2016; Khan et al., 2014; Liu et al., 2014; Selph and Carson, 2011) and with the most common organisms responsible for orthopaedic SSIs (Hickok and Shapiro, 2012). However, based on the present and others’ *in vitro* data, reduction in bacterial adhesion and biofilm formation requires high levels of antibiotics (Costerton et al., 1999), levels becoming even higher when serum proteins are present (Gilbertie et al., 2019). Thus, the study sought what effects antibiotic concentrations in these ranges would have on adherent bacteria cultured in fluids derived from physiological environments, *i.e.* SynF and serum.

Pre-formed biofilms were used to model implant contamination such as would be observed in established infections. In keeping with many other studies (Dastgheyb et al., 2015a; Donlan and Costerton, 2002; Mandell et al., 2019), these biofilms were tolerant to antibiotics both in TSB and SynF. While the biofilm used was relatively immature (24 h), even these biofilms exhibited marked insensitivity, underlining the difficulty in decreasing numbers of already adherent bacteria. It has been suggested that a 48 h biofilm would better model *in vivo* implant contamination and hence treatment strategies (Baeza et al., 2019). Therefore, the limited success with antibiotics observed in the present study might be even less when testing these more mature biofilms, which can be considered as modelling established infections. Another aspect to consider is the known time-dependent effects of VAN on bacterial eradication (Post et al., 2017). It is not clear how this property will impact bacteria exposed to the rapidly decreasing VAN, as measured in the sheep model, especially as the 100-200 mg/mL VAN was maintained for more than 7 d in Post et al. (2017) study. It will be important to determine if this time-dependent killing can be exploited *in vivo* to enhance eradication of adherent bacteria.

Faced with the difficulty in eradicating established biofilms, prevention of bacterial adhesion, as exemplified by the antibiotic prophylaxis that occurs concomitantly with perioperative contamination, becomes a focus. Of clinical importance is the fact that bacterial adhesion takes place within minutes to hours (Hall-Stoodley et al., 2004; Saeed et al., 2019), and longer antibiotic prophylaxis regimens may not significantly alter infection rates (Bondarenko et al., 2019). The current study was aimed at determining conditions where a BPC (Macia et al., 2014) of antibiotics may be reached.

The present *in vitro* BPC model, in which bacteria were added simultaneously with antibiotics, showed that 5× MIC VAN decreased bacterial adherence in all media but 50× MIC was required in TSB and 250× MIC in SynF (Fig 6). Using GEN, 40× MIC was sufficient to reach a 2 log decrease in average bacterial counts (Fig 7b) but, even in the presence of 400× MIC GEN, bacterial adhesion was still measured. Importantly, the presence of serum proteins in the 50 % HS or SynF samples attenuated the effectiveness of the antibiotics. While the *in vitro* investigations were limited in scope, VAN was clinically demonstrated to be an effective local treatment while the data for GEN was less clear (Lin et al., 2021). The present data may support reservations for the use of GEN as a solo treatment.

Because GEN elution systems are used to supplement perioperatively administered VAN, the effect of staged addition of the two antibiotics was studied. VAN and aminoglycosides such as GEN have been combined and both no synergy (Streuli et al., 2006) and synergy (McGowan, 1998) reported against Staphylococcal species. There have been mixed results as to whether VAN and GEN, as well as other antibiotic combinations with VAN, show synergism, in particular against MRSA (Deresinski, 2009; Mulazimoglu et al., 1996). Interestingly, a staged addition of the aminoglycoside streptomycin with the cell-wall active penicillin enhances efficacy against planktonic *Escherichia coli* (Davis, 1982). VAN and amikacin, another aminoglycoside, have demonstrated synergism against MSSA in the planktonic form, but not when biofilm embedded (Broussou et al., 2018). Thus, whether the cell-wall-targeted VAN would synergise with the aminoglycoside GEN against planktonic and adherent MSSA was tested. In a checkerboard assay, additive effects were observed against planktonic bacteria, independent of order or timing of addition. When adherent bacteria were analysed, concomitant addition of GEN + VAN was not additive and furthermore not statistically different from controls. Staged delivery showed trends towards enhanced activity, where 20 min gaps consistently showed statistical significance against the control as well as concomitant addition of GEN + VAN. These staged addition studies, while only explored in TSB, suggested additional strategies, albeit translation to a clinically realistic protocol may be challenging.

There were limitations to the present study. MSSA ATCC^®^ 25923^™^, a widely used, biofilm-forming reference strain (Treangen et al., 2014), was used for determination of the antibiotic effects. Findings would be more generalisable with additional strains of MSSA as well as coagulase-negative Staphylococci. Another limitation aroses in the use of different media. The ideal medium TSB was used as well as media rich in serum proteins, *i.e.* SynF aspirated from the joint (Felgenhauer and Hagedorn, 1980) or diluted HS as a surrogate for wound fluid (Buchan et al., 1981; Cutting, 2003; Katz et al., 1991). The commonality of serum proteins between the two fluids provided a framework for the proteinaceous bacterial matrix characteristic of *in vivo* bacteria aggregates and biofilms. The impact of these fluids was tested and while different, the antibiotic sensitivity of MSSA in HS and SynF was markedly reduced, although not equivalent. Based on the presence of serosanguinous fluid immediately post operatively, even in the joint, (whereas SynF would be more characteristic of longer times), and the clear increase in antibiotic resistance with the presence of serum proteins, the study was carried on using the more easily sourced 50 % serum. These fluids derive from tissue environments and, while more faithful to *in vivo* conditions, do not replicate the native cells nor the immune-cell-rich environment of the tissue (Spear, 2012). Also, serum binding of drugs was not measured. Importantly, *in vivo* results demonstrated a dynamic antibiotic concentration profile, whereas *in vitro* experiments maintained the designated antibiotic concentration for the duration of the experiment. *In vitro* biofilm characteristics, such as growth age and exposure time (Chen et al., 2020) as well as timeline of antibiotic therapy (Post et al., 2017), are important to consider when evaluating *in vitro* results (Chen et al., 2020). *In vivo*, Castaneda et al., (2016) demonstrated that a 5 d course of antibiotic exposure lower the MBEC compared to a 24 h exposure, suggesting that a 24 h model may overestimate minimum concentrations needed to eradicate biofilms *in vivo*. Therefore, *in vitro* experiments using constant, high antibiotic concentrations for 24 h may underestimate antibiotic requirements *in vivo* where falling concentrations confound the antibiotic effects.

Those findings raise questions about the effect of local concentrations of antibiotics against contaminating bacteria. The data obtained suggested that even perioperative sterilisation of surrounding tissue or implant surfaces would be quite difficult, indicating that additional study of both *in vitro* and *in vivo* systems is needed, especially in the context of the applicability of MIC to therapeutic outcomes.

## Discussion with Reviewer

### David Grainger:

Time points *in vitro* for drug exposure to pathogen and pathogen growth on surface are not clearly correlated to known *in vivo* infection susceptibility or implant infection time course. How might this be better incorporated into the study design?

### Authors:

The implant infection time course is widely accepted to start with contamination of the implant site during surgery. The time course of biofilm formation *in vivo* is not as clearly defined as that *in vitro*. However, at a recent discussion at the HSS Biofilm Conference (Hospital for Special Surgery, New York City, 2018), the physicians ascribed to the idea that the *in vitro* timeline was in fact quite applicable. Importantly, we are focused on biofilm prevention as represented by adhesion in the presence of antibiotics and of antibiotic treatment of early biofilms. The lack of efficacy of very high levels of VAN against even 24 h old biofilms showed the dire need for preventing biofilm formation in the first place. The present *in vitro* models, while not without limitations, provide valuable insight into what it takes to prevent bacterial colonisation of implant surfaces.

### David Grainger:

How might antimicrobial effects (*e.g.* changing MIC) *in vitro* be better considered and compared to those *in vivo* given physiological drug-plasma binding and pharmacokinetic/pharmacodynamic effects not present *in vitro*?

### Authors:

The main strength of the *in vitro* investigations was that the dilutions of fluids used were in the range of those surronding an orthopaedic implant. These media would exhibit similar physiological drug-plasma binding and would assist biofilm formation by way of serum proteins. Of course, the key missing component is the complexity of the site, including the tissue and fluid flow. Although there are tissue-on-a-chip constructs that more exactly mimic the equilibrium between the many different components, these are minor aspects in comparison to the *in vivo* situation and the information represented in pharmacokinetic/pharmacodynamic determinations. It is the authors’ opinion that the use of fluids rich in serum proteins at least mimics the first step in the procedure: the interaction of the drugs with the proteins in the milieu. Fortunately, such studies have been fruitful for understanding antibiotic effects, despite their limitations.

## List of Abbreviations

ANOVA: analysis of variance
ARRIVE: Animal Research: Reporting of *In Vivo* Experiments
AUC: area under the curve
BPC: biofilm-prevention concentration
CFU: colony-forming unit
Cmax: maximum concentration
DAIR: debridement antibiotics and implant retention
DI: deionised
ECF: extracellular fluid
ECG: electrocardiogram
EDTA: ethylenediaminetetraacetic acid
eqSynF: equine SynF
FIC: fractional inhibitory concentration
FIO_2_: fraction of inspired oxygen
GEN: gentamicin
HPLC: high-performance liquid chromatography
HS: human serum
IACUC: Institutional Animal Care and Use Committee
IM: intramedullary
LC/MS/MS: liquid chromatography with tandem mass spectrometry
LCP: locking compression plate
MBEC: minimum biofilm eradication concentration
MHB: Mueller Hinton broth
MIC: minimal inhibitory concentration
MRSA: methicillin-resistant *S. aureus*
MSSA: methicillin-sensitive *S. aureus*
ON: overnight
PBS: phosphate-buffered saline
PJI: prosthetic joint infection
PLA: poly lactic acid
PMMA: polymethyl methacrylate
PO: periosteal
RT: room temperature
*S. aureus*: *Staphylococcus aureus*
SEM: scanning electron microscopy
SQ: subcutaneous
SSI: surgical site infection
SynF: synovial fluid
Tmax: time to max concentration
TSB: trypticase soy broth
VAN: vancomycin
